# Assessing the integrity and mechanical properties of commercial microneedles: innovation or fad?

**DOI:** 10.1007/s13346-025-01888-8

**Published:** 2025-05-31

**Authors:** Jing Yi Lee, Shi Hui Dong, Keng Wooi Ng, Choon Fu Goh

**Affiliations:** 1 Discipline of Pharmaceutical Technology, School of Pharmaceutical Sciences, Universiti Sains Malaysia, Minden, Penang, 11800 Malaysia; 2 School of Pharmacy, Faculty of Medical Sciences, Newcastle University, Newcastle upon Tyne, NE1 7RU UK; 3 Translational and Clinical Research Institute, Faculty of Medical Sciences, Newcastle University, Newcastle upon Tyne, NE1 7RU UK

**Keywords:** Microneedles, Needle dimension, Product integrity, Packaging, Skin puncture

## Abstract

With the rapid advancements in microneedle technology, the translation of microneedles to end users is now achievable. However, concerns about microneedle product quality persist due to the absence of standard guidelines. This study aims to evaluate the integrity, mechanical properties and skin penetration performance of commercially available polymeric-based cosmetic microneedle patches. The patches were characterised for needle geometry/dimension, mechanical strength and insertion capabilities using in vitro Parafilm^®^ M and ex vivo porcine skin models. Ten microneedle patches investigated have needle height of ~ 180–500 μm, base width of ~ 100–270 μm and interneedle spacing of ~ 320–686 μm with fracture force up to 14 N. Some microneedles showed deformed needles due to poor packaging protection. Despite most microneedle patches showed good insertion into Parafilm^®^ M, only 7 patches demonstrated ex vivo porcine skin insertion in OCT analysis. Nevertheless, only 1 microneedle patch demonstrated full skin insertion ratio due to a lower needle density. This study highlights the critical role of needle geometry, mechanical properties and packaging in ensuring microneedle functionality. More importantly, microneedle products are a significant ‘innovation’ that must prioritise puncture performance and packaging to ensure effectiveness and avoid being dismissed as a mere ‘fad’.

**Graphical abstract:**

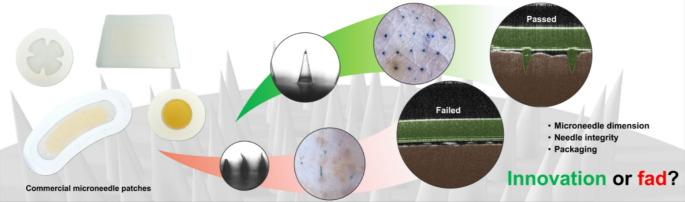

**Supplementary Information:**

The online version contains supplementary material available at 10.1007/s13346-025-01888-8.

## Introduction

Microneedles (MN) have emerged as a cutting-edge, minimally invasive approach to delivering drugs into the skin in recent decades. The global market for MN drug delivery system is anticipated to expand significantly, increasing from USD 6.1 billion in 2024 to USD 9.5 billion by the close of 2030, reflecting a compound annual growth rate of 7.7% [1]. In 2024, North America led the global MN market, holding a 43.1% of revenue share, followed by Europe and the Asia Pacific region. This rapid growth underscores the increasing demand of MN patches, especially due to their ability to facilitate painless self-administration.

Henry, McAllister, Allen and Prausnitz [2] documented the first successes of MN-mediated drug delivery in their seminal work even though there had been several prior patents [3–5]. Since the successful case, many possibilities have emerged that could not have been realised using conventional formulations including delivering drug molecules with large molecular weight (> 500 Da) such as amphotericin B [6] and rapamycin [7] and low aqueous solubility (or insoluble) such as elemene [8] as well as proteins and peptides such as hormones [9], insulin [10] and vaccines [11, 12]. This breakthrough has propelled both research and commercialisation of MN technology. Prausnitz and Langer [13] described MN as third-generation transdermal delivery systems which substantially enhance skin permeability by creating a higher degree of skin disruption that enables the delivery of macromolecules such as proteins and vaccines.

Several MN-based pharmaceutical products have been marketed for vaccine delivery, including Intanza^®^ in Europe (later withdrawn) and Fluzone^®^ in US for intradermal administration of trivalent inactivated split-virion influenza vaccine [14–18]. These technologies are mainly hollow MN intradermal injection devices (Soluvia^®^ prefilled glass syringe) featuring only a single micron-sized needle (30-gauge microneedle with short-bevel shape that extends 1.5 mm from a depth-limiting tip) [18]. Nevertheless, some may not perceive these products as true MN products but just an intradermal needle injection. On the other hand, microneedling is more popular in cosmetic purposes such as skin rejuvenation, scar and wrinkle reduction where it is widely used to diminish wrinkles and stimulate collagen production due to the micro-wounds created [19]. To pave way for the commercialisation of this innovative technology, polymeric MN patches are poised for significant market growth owing to their ability to incorporate actives into the patch and allow for easy self-administration with a single step. Despite their strong promise in (trans)dermal delivery, the commercialisation of polymeric MN remains a huge challenge due to the complexities involved in manufacturing and the high regulatory cost.

For cosmetic MN patches, the global beauty market for mainly dissolving MN type is valued at USD 0.67 billion in 2024 but it is projected to grow faster at a compound annual growth rate of 12.8% to USD 1.56 billion by 2032 [20, 21]. Furthermore, polymeric MN patches for cosmetic use have recently gained considerable traction, especially in Asian markets. Perhaps owing to lower regulatory hurdles, the commercial development of polymeric MN products for cosmetic use has outpaced pharmaceutical MN products for a long time. Indeed, cosmetic MN products are not only generally available in local pharmacies but have also been easily accessible through e-commerce platforms for over a decade. These cosmetic dissolving MN products in the current market are generally found to contain active compounds such as vitamin C, hyaluronic acid and various plant extracts, targeting for a wide range of indications including but not limited to anti-wrinkle, whitening and anti-acne actions [20, 22]. Valuable lessons from the commercialisation of cosmetic MN products can be leveraged to inform the commercial development of MN products for pharmaceutical applications.

However, while the polymeric-based cosmetic MN products have generated significant consumer interest, there has been a lack of scientific reports in the public domain regarding their designs and effectiveness at skin penetration. Therefore, this work seeks to bridge that knowledge gap by systematically evaluating the effectiveness of commercially available cosmetic MN product designs, including the relationship between MN dimensions, mechanical properties, product packaging and skin penetration efficiency. By sharing our findings in the public domain, we hope to help expedite commercialisation efforts for pharmaceutical MN products.

## Materials and methods

### Materials

Ten cosmetic MN patches were randomly purchased from e-commerce platforms and local stores based on the appropriate product descriptions on the packaging that matched the MN criteria. Parafilm^®^ M, a flexible thermoplastic sheet (110.0 ± 1.2 μm; *n* = 5; different locations of Parafilm^®^ M sheets) made of olefin-type material, was obtained from Brand GmbH (Wertheim, Germany). Porcine ear skin was purchased from a local slaughterhouse in Penang, Malaysia. The hair was plucked off carefully and the underlying fat layer was removed. The excised skin was stored at -20 °C and thawed at room temperature before use.

### Characterisation of MN patches

#### Microscopic analysis

**Fig. 1:**
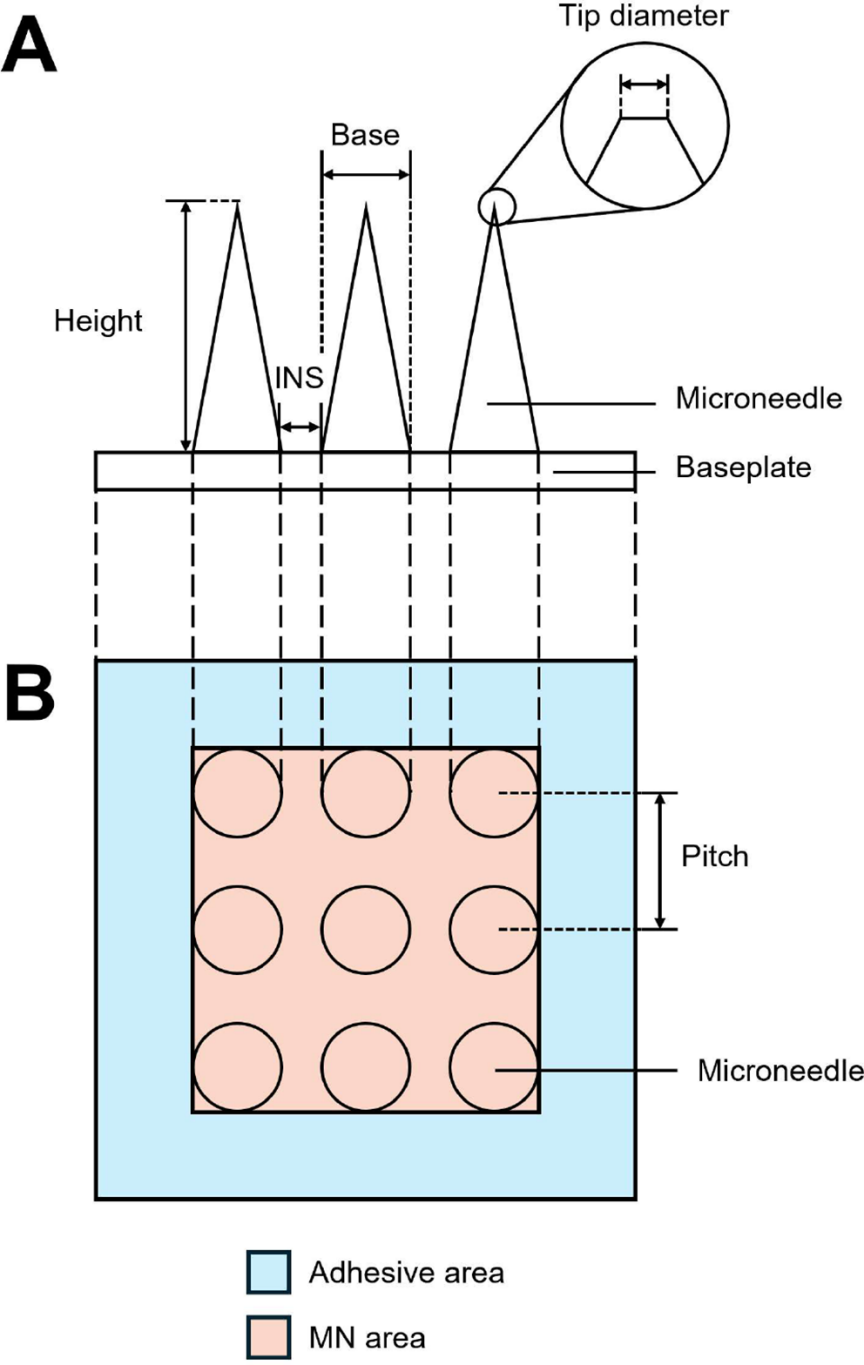
Schematic illustration of dimensional measurements of MN patches in (**A**) side view and (**B**) top view

The MN patches were visualised under an optical microscope (BX53, Olympus, Tokyo, Japan) fitted with a DP72 microscope digital camera at a magnification of 6.3×. The geometry and dimensions (height, base, interneedle spacing (INS) (edge-to-edge of needle base), pitch (centre-to-centre distance between needles), MN distribution pattern and tip diameter) of MN patches were determined using the cellSens Standard Imaging software (1.4.1 (Build 8624) Olympus Corporation, Tokyo, Japan) as illustrated in Fig. 1. The MN aspect ratio was calculated as the ratio of the MN height to the MN base width. The patch area (area covering the whole patch including the adhesive part) and MN array area (area only containing the needles excluding the adhesive part) were estimated using ImageJ software (version 1.54p, National Institutes of Health, Bethesda, MD, US) while the needle density was calculated as shown in Eq. (1). The measurements were taken from 3 patches per product, at 15 needles per patch.

1 $$\:Needle\:density=\:\frac{Number\:of\:needles}{MN\:array\:area\:\left({mm}^{2}\right)}$$

#### Mechanical testing of MN patches

##### Axial compression test

The mechanical properties of MN patches were determined using Texture Analyser TA-XT (Stable Micro Systems, Surrey, UK) [23]. A metal cylindrical probe (diameter: 50 mm; P/50) was lowered onto the MN patches adhered to the platform with double-sided tape at a pre-test speed of 0.1 mm/s. The compression test was performed in distance mode with a rate of 0.1 mm/s and a total displacement of 0.5 mm, followed by a post-test speed of 0.5 mm/s. The force and displacement data were used to quantitatively determine the fracture force of MN patches with a sudden drop in the force as the needle failure force [23].

##### MN height reduction test

Under the same setup, the MN height reduction test was using the same Texture Analyser as described in the section Axial compression test. Under compression mode, the probe was set to move at a test speed of 0.1 mm/s until the required force (32 N) was exerted with a trigger force of 0.049 N [23]. The forces was held for 10 s and the probe was moved upwards at a speed of 0.5 mm/s. The MN patches were then visualised under the optical microscope after the compression as described in the section Microscopic analysis. The ability of MN patches to withstand the compression was also expressed in terms of percentage MN height reduction, calculated based on Eq. (2).

2 $$\begin{aligned}\eqalign{& \>Percentage\>of\>MN\>height\>reduction\>\left( \% \right) \\& \qquad= {\matrix{ Initial\>MN\>height - Final\>MN\>height \hfill \cr} \over {Initial\>MN\>height}} \times \>100\% \cr} \end{aligned}$$

#### Membrane insertion test in skin models

##### Parafilm^®^ M insertion study

The insertion efficiency of MN patches was evaluated using Parafilm^®^ M, a skin simulant as described [23, 24]. Briefly, the Parafilm^®^ M was cut according to the size of the patches and stacked to obtain an eight-layer film with ~ 1 mm thickness (~ 110 μm per layer). The stacked Parafilm^®^ M layers were then placed onto a sheet of dental wax over a corkboard for support. The MN patches were manually pressed by applying thumb force (~ 20 N) for 30 s. The MN patch was removed from the Parafilm^®^ M layer and the insertion capability was expressed as the number of holes in each layer. The percentage of holes in Parafilm^®^ M layer was calculated as shown in Eq. (3).

3 $$\begin{aligned}& \text { Percentage of holes in Parafilm }{ }^{\circledR} \text { M layer (\%) } \\& \qquad=\frac{\text { Number of holes in Parafilm }{ }^{\circledR} \text { M layer }}{\text { Total MN number }} \times 100 \%\end{aligned}$$

##### Ex vivo porcine skin insertion study

Using a similar setup as the Parafilm^®^ M insertion study (see section Parafilm^®^ M insertion study), the skin insertion efficiency of MN patches was investigated using ex vivo porcine ear skin [23]. Porcine ear skin was used due to its close structural resemblance to human skin, including comparable hair follicle density, stratum corneum (SC) thickness, stratified multilayered, keratinising epithelium and viable epidermis thickness [25, 26]. The MN was manually inserted into the excised skin with thumb force (~ 20 N) for 30 s. The MN patch was then removed. A few drops of methylene blue solution were added on to the pierced skin for 30 min. The stained skin was tapped dry with paper towel to remove excess dye. The ability of the MN patches to penetrate the SC was evaluated by counting the blue-stained spots on the skin surface. The skin insertion ratio was determined using Eq. (4). In addition, the experiment was repeated using in situ optical coherence tomography (OCT; Lumedica LabScope 2.0, Edmund Optics, York, UK). To allow differentiation between the MN and the skin layers, false colours were applied using Microsoft Powerpoint. The MN patches were observed and captured before and after the OCT analysis using a CETI Steddy-T stereomicroscope (Medline Scientific, Oxon, UK) equipped with an 18-megapixel D18 digital camera and ToupLite software (Medline Scientific, Oxon, UK).

4 $$\begin{aligned}\eqalign{&\>Skin\>insertion\>ratio\>\left( \% \right) \\ & \qquad= {\matrix{ Number\>of\>blue- stained\>spots\>on\>the\>skin \hfill \cr} \over \matrix{ Total\>MN\> Number \hfill \cr} } \times \>100\% \cr} \end{aligned}$$

### Statistical analysis

All statistical tests were performed via IBM SPSS Statistics version 29.0 (IBM Corp., Armonk, NY, US). The skewness and kurtosis of data were monitored to determine the data distribution. Parametric data typically showed a normal distribution with the skewness and kurtosis between − 1 and + 1. Otherwise, the data were considered non-parametric. Pearson’s correlation was performed to investigate the relationship between dimensional measurements, mechanical properties and insertion profiles. Multiple regression analyses (MRA) were carried out to further elucidate the effect of dimensional measurements on mechanical properties and insertion profiles of MN patches. Due to the limited data, the analysis was performed by sequential exclusion of parameters that did not contribute statistically significantly until a statistically significant regression model was obtained. All data were expressed as mean ± SD and *p* value of < 0.05 was considered statistically significance in all cases.

## Results & discussion

### Content and packaging of MN patches

**Fig. 2:**
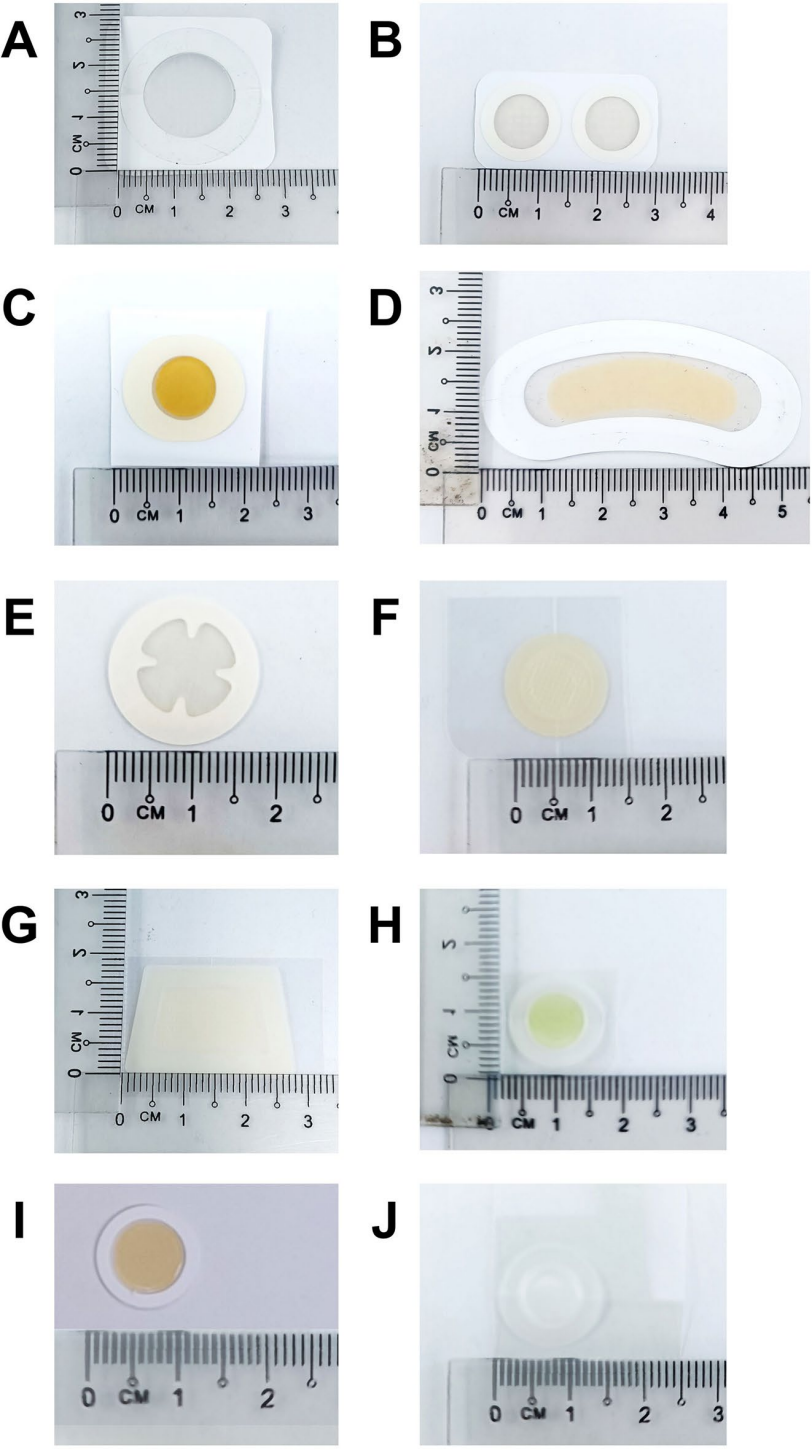
Photographs of MN patches (MN-A – MN-J)

Figure 2 shows the photographs of the MN patches while their content, dimension, indications and other relevant information obtained from product packaging and websites are summarised in Table 1. The products have been anonymised to protect potentially sensitive commercial information. Nearly 70% of the MN patches (MN-A, MN-B, MN-E, MN-F, MN-G, MN-H and MN-J) were manufactured in South Korea, with the remainder sourced from China (MN-C, MN-D and MN-I). Surprisingly, none of the MN patches carried the term ‘microneedle(s)’ in the product name. Instead, the majority of MN patches (MN-A, MN-B, MN-D, MN-E, MN-G, MN-H and MN-I) use terms like ‘microdart(s)’, ‘microcone(s; a registered trademark)’, ‘microdot(s)’, ‘micropoint(s)’ or ‘microshot(s)’ on their packaging (data not shown). Only MN-B showed the term ‘microneedle(s)’ on the product website. These terms were likely chosen because the term ‘needle’ may evoke negative perceptions among consumers such as association with sharpness, pain and bleeding, potentially leading to misinterpretations and reduced product acceptance in the market [27]. Furthermore, in some countries, including the word ‘needle’ in the product name may classify it as a medical device rather than a cosmetic product, making product registration easier. About 60% of the products (MN-A, MN-C, MN-F, MN-H, MN-I and MN-J) contained tea tree oil, *Centella asiatica* extract and/or witch hazel extract which are indicated for acne and sebum control [28]. While the other MN patches, which were advertised for whitening and brightening (MN-B, MN-D, MN-E and MN-G), contained niacinamide, ascorbic acid or glutathione. Most MN patches were advised to be applied overnight (6–8 h) as stated on their product packaging. The recommended application sites were indicated based on their intended use. For instance, MN patches for acne and sebum control were advised to be applied directly on the acne spots. While those patches indicated for whitening and brightening were recommended to be used on the desired facial areas such as cheeks or under-eye regions. These usage instructions may reflect the targeted delivery needs and site-specific actions of the active compounds embedded within the MN patches. All MN patches included a hydrocolloid dressing probably as a baseplate material which functions to absorb pus and exudate, especially in acne management.

**Table 1:** Product description of MN patches investigated based on the product packaging and information on the product website

Code	Active ingredients	Other ingredients	Patch shape	Overall patch dimension* (mm)	Estimated MN array area** (mm^2^)	Number of patches/ product	Indications/ target effects	Instruction of application	Recommended application period	Recommended application site	Country of origin
MN-A	*Hamamelis virginiana* (witch hazel) extract, panthenol, *Epilobium fleischeri* extract, adenosine, *Taraxacum officinale* (dandelion) leaf extract, acetyl octapeptide-8 and pomegranate extract	Sodium hyaluronate, butylene glycol and 1,2-hexanediol	Circle	26 (*d*)	201	4	Reduce pore size and sebum production	Apply on dry and cleansed skin	≥ 2 h	Nose and cheek	South Korea
MN-B	Arginine, oligopeptide-76, palmitoyl oligopeptide, niacinamide and ferulic acid	Sodium hyaluronate and sodium hydroxide	Circle	14 (*d*)	64	6	Pore cleaning and skin soothing effect	Press patch vertically with thumb	< 2 h	Acne site	South Korea
MN-C	Tea tree oil and hyaluronic acid	Hydrocolloid	Oval	19 (*h*) × 16 (*w*)	64	9	Acne treatment	Apply patch vertically for 5–10 s	6–12 h	Acne site	China
MN-D	Niacinamide, psoralen powder and hyaluronic acid	Xanthan gum and ethylhexylglycerin	Bean	48 (*h*) × 16 (*w*)	192	2	Dark circles and puffiness reduction effect	Apply on dry and cleansed skin	10 min	Under-eye area	China
MN-E	Niacinamide, glutathione (20,000 ppm), ethyl ascorbyl ether and riboflavin	Sodium hyaluronate, trehalose, glycerin, ectoin, 1,2-hexanediol and ethylhexylglycerin	Circle	18 (*d*)	154	12	Skin whitening effect	Wash and dry affected area. Press firmly from the centre of the patch	≥ 4 h	Pigmentation site	South Korea
MN-F	*Hamamelis virginiana* (witch hazel) extract, C*amellia sinensis* leaf extract, G*lycyrrhiza glabra* (licorice) root extract, madecassoside, *Melaleuca alternifolia* (tea tree) leaf oil, beta glucan and salicylic acid	Sodium hyaluronate, cellulose gum, glycerin, trehalose, propanediol, 1,2-hexanediol, caprylyl glycol and ethylhexylglycerin	Circle	14 (*d*)	64	9	Acne treatment	Apply on dry and cleansed skin. Press and hold for 10 s	6–8 h	Acne site	South Korea
MN-G	Ascorbic acid (50,000 ppm), glutathione (25,100 ppm), niacinamide, tranexamic acid (60,900 ppm), ferulic acid and tranexamoyl dipeptide-23	Sodium hyaluronate, cellulose gum, trehalose, 2,3-butanediol, propanediol, caprylyl glycol, butylene glyol, ethylhexylglycerin and 1,2-hexanediol	Trapezoid	27.6 (*h*) × 18 (*w*)	165	6	Skin brightening effect	Gently apply the patch and press it until it adheres to the skin	6–8 h	Pigmentation site	South Korea
MN-H	*Carthamus tinctorius* (safflower) flower extract, *Gardenia florida* fruit extract, panthenol, salicylic acid, *Melaleuca alternifolia* (tea tree) leaf oil, madecassoside, C*entella asiatica* extract, C*amellia sinensis* leaf extract, beta glucan and C*amellia sinensis* catechins	Sodium hyaluronate, butylene glycol, trehalose, dextrin, dimethyl sulfone, dipotassium glycyrrhizate, 1,2-hexanediol and glycerin	Circle	14 (*d*)	64	9	Acne treatment and hyperpigmentation	Apply on dry and cleansed skin. Press and hold for 3 s	< 12 h	Acne site	South Korea
MN-I	Tea tree oil, *Centella asiatica* extract, purslane extract, glycerin and dogwood extract	Not stated	Circle	9 (*d*)	28	2	Acne treatment	Apply on dry and cleansed skin	< 8 h	Acne site	China
MN-J	Madecassoside, 4-terpineol, *Melaleuca altemifolia* (tea tree) leaf oil, *Salix alba* (willow) bark extract, *Brassica oleracea gemmifera* (brussels sprouts) extract, *Brassica oleracea italica* (broccoli) extract, prunus mume extract, betaine salicylate, glycolic acid and *Polygonum cuspidatum* root extract	Sodium hyaluronate, glycerin, trehalose, polyglycerol-10 laurate, caprylic/capric triglyceride, propanediol, butylene glycol, myristyl alcohol, protocatechuic acid and 1,2-hexanediol	Circle	14 (*d*)	38	9	Sebum reduction	Apply on dry and cleansed skin. Press and hold for 3 s	8–10 h	Acne site	South Korea

*d: diameter; *h*: height; *w*: width; **: the area only containing the needles (excluding the adhesive part)

Additionally, 90% of the MN patches were packed in sets of ≤ 9 saved for MN-E containing 12 MN patches per set as listed in Table 1. For the MN patch design, 80% of them have a circular shape with exceptions for MN-D (bean-shaped) and MN-G (trapezoid). The circular MN patches have an estimated MN array area of 28 mm^2^ (MN-I) – 201 mm^2^ (MN-A) while MN-D and MN-G have among the largest sizes of 192 and 165 mm^2^, respectively, due to their unique shape. Despite differences in shape and size, all MN patches fit within the average size of the human thumb pad (34.5 mm in height and 23 mm in width) for an appropriate application of MN on the skin [29, 30].

The variations in shape, size and quantity of patches per product may be a careful consideration for matching the targeted indications. Circular-shaped MN patches are commonly found to be supplied in a higher patch number (MN-C, MN-E, MN-F, MN-H and MN-J) which may be related to their indication for acne and sebum control due to better coverage of the acne spots. Conversely, the unique shapes of MN-D and MN-G with their larger size may be specially designed to match the facial contours, such as the under-eye region, or to accommodate larger surface areas for other skincare functions such as skin whitening. Nevertheless, these design choices may influence user comfort and adherence, especially for extended applications on the sensitive facial areas.

Table 2 shows the packaging types of all MN patches. It is important to have secure packaging for MN patches due to possible damage during transportation or handling. All MN patches came with primary packaging but only 80% (MN-A, MN-B, MN-C, MN-E, MN-F, MN-G, MN-H and MN-J) included both primary and secondary packaging. Various types of primary packaging were used, such as plastic boxes with or without individual compartments (MN-A, MN-C, MN-F, MN-G, MN-H and MN-J), aluminium-plastic peelable blister packaging (MN-B and MN-E) and paper boxes (MN-D and MN-I). Peelable blister packaging and plastic boxes with individual compartments, as demonstrated by MN-A, MN-B and MN-E, may offer better stability to preserve MN integrity during handling and storage. In plastic boxes without individual compartments (MN-C, MN-F, MN-G, MN-H and MN-J), a plastic film was used to hold multiple MN patches in place and prevent them from moving or sticking to each other. However, MN patches packaged in paper boxes may lack tamper-evident protection, potentially compromising product integrity due to the direct exposure of needles to the box or mishandling. There are only two MN patches (MN-D and MN-I) with paper boxes as the only packaging. 

**Table 2:** Types of packaging used for MN patches

Code	Primary packaging		Secondary packaging		
	Presence	Type	Presence	Type	Silica gel
MN-A	✓	Plastic box with individual compartments	✓	Non-resealable plastic bag with aluminium foil lining and paper box	×
MN-B	✓	Aluminium-plastic peelable blister package	✓	Resealable plastic bag with aluminium foil lining and paper box	×
MN-C	✓	Plastic box with plastic film separation	✓	Resealable plastic bag with aluminium foil lining and paper box	✓
MN-D	✓	Paper box	×	×	×
MN-E	✓	Aluminium-plastic peelable blister package	✓	Paper box	×
MN-F	✓	Plastic box with plastic film separation	✓	Resealable plastic bag with aluminium foil lining	✓
MN-G	✓	Plastic box with plastic film separation	✓	Resealable plastic bag with aluminium foil lining	✓
MN-H	✓	Plastic box with plastic film separation	✓	Resealable plastic bag with aluminium foil lining	×
MN-I	✓	Paper box	×	×	×
MN-J	✓	Plastic box with plastic film separation	✓	Resealable plastic bag with aluminium foil lining	✓

Notably, secure secondary packaging consisting of a plastic bag with an aluminium foil lining, either with (MN-C, MN-F, MN-G, MN-H and MN-J) or without zip-lock features (MN-A), was also employed. Additionally, three of those with a zip-lock plastic bag (MN-F, MN-G and MN-J) were also provided with silica gel. The MNs with resealable packaging and silica gel are anticipated to exhibit better product stability over time with the moisture control provided. According to the list of Critical Quality Attributes (CQA) established by a Regulatory Working Group under PATH’s Centre of Excellence [31, 32], the physical attribute of ‘container close system/packaging’ was placed in the least ‘critical’ and ‘prioritised’ quadrant of MN products [27]. However, another closely related physical attribute, physical stability (integrity before application), was ranked in the highest quadrant, highlighting the crucial role of packaging in maintaining needle integrity and functionality from production to consumer use. The importance of primary MN packaging in providing a moisture barrier function and temperature resistance has also been emphasised by McAlister, Kearney, Martin and Donnelly [33] to ensure the stability of MN patches during storage, transport and distribution across different climatic regions.

### Microscopic images and dimension analysis of MNs

**Fig. 3:**
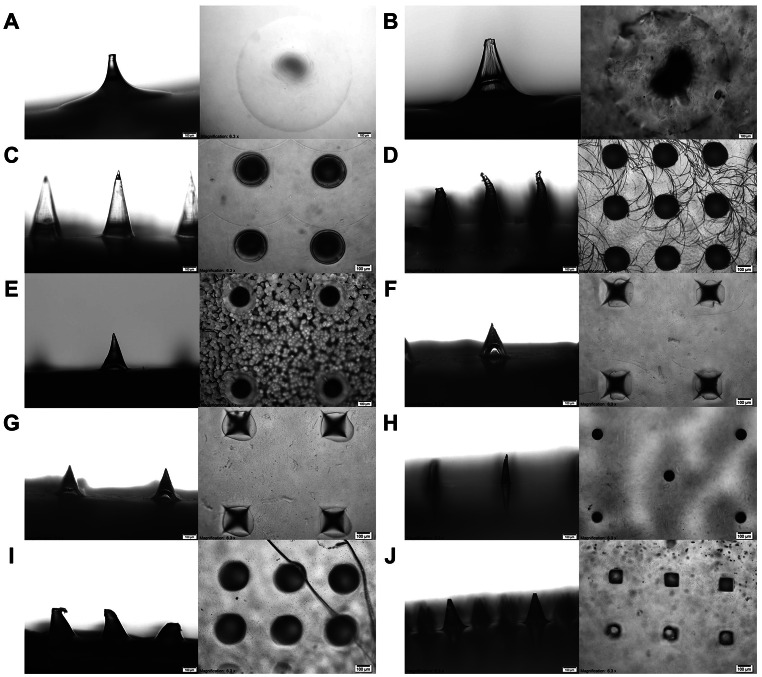
Microscopic images of MN patches (alphabet represents MN code) from the lateral (left panel) and top (right panel), at a magnification of 6.3× with a scale bar of 100 μm

Figure 3 shows the lateral and top views of MN patches under light microscopy. Several studies have highlighted the needle geometry is a critical factor for drug loading, stress distribution and mechanical properties, which ultimately influence the penetration and dissolution of MNs in the skin [34, 35]. Most MN patches were conical while the rest (MN-F and MN-G) were pyramidal. It has been collectively reported that conical MNs offer a higher volume and a larger drug loading capacity compared to those with cone-cylinder, rectangular pyramid and hexagonal pyramid shapes [34, 35]. MNs with different base shapes (e.g. triangle, square, hexagonal and star) have been found to achieve deeper penetration due to the edge action on the skin during insertion [36, 37].

Based on the needle geometry, MN-A and MN-B were probably fabricated via the drawing lithography, where 3D MN structures are directly extended from a 2D viscous polymer material [38]. While the remaining MN patches had a well-defined shape, which could have been produced using a micromolding technique. It is interesting to note that most of the needles were intact, except MN-D and MN-I, in which the needles were found to be bent and deformed (Fig. 3D and I) due to insufficient protective packaging. These observations give an initial indication about the importance of packaging for ensuring MN integrity, which may have a strong influence on their mechanical properties and skin insertion.

Table 3 tabulates the needle dimension measurement across all MN patches. Most MN patches in the study have ≥ 100 needles per patch except MN-A (75 needles) and MN-B (16 needles). Almost all MNs have a needle height of ~ 200–400 μm except MN-C, which had the greatest height of 504.98 ± 10.39 μm. While MN-A recorded the largest base width at 879.11 ± 10.96 μm, the rest of the patches only had a base width of ~ 100–270 μm.

The aspect ratio can affect the ease of skin insertion and mechanical durability of a needle. The aspect ratio of MN has been variously defined as the ratio of base to tip diameter [39], ratio of MN height to tip diameter [40] and the ratio of pitch to MN radius [41]. In this work, the aspect ratio is defined as the height-to-base width ratio as reported by Wilke, Mulcahy, Ye and Morrissey [42] and Davis, Martanto, Allen and Prausnitz [43]. Needles with a higher aspect ratio are expected to penetrate the skin more easily, whereas those with a lower aspect ratio offer greater mechanical strength [44]. An aspect ratio of 2:1 and above is widely adopted as the standard for mechanically robust MN [45]. However, all MN patches purchased in the current study had an aspect ratio of < 2:1. Despite of the rule of thumb, we anticipated that these MN patches would still demonstrate adequate mechanical strength.

**Table 3:** Dimensional measurement of MN (*n* = 5; mean ± SD)

Code	Number of needles per patch	Height (µm)	Base (µm)	Tip diameter (µm)	Aspect ratio	INS* (µm)	MN distribution pattern	Pitch** (µm)	Needle density
MN-A	75	362.86 ± 11.08	879.11 ± 10.96	50.36 ± 6.36	0.42 ± 0.1	433.08 ± 12.8	Square	1301.98 ± 7.02	0.38
MN-B	16	280.68 ± 7.49	262.04 ± 6.6	6.46 ± 2.31	1.08 ± 0.1	685.6 ± 12.2	Square	1335.9 ± 29.77	0.25
MN-C	170	504.98 ± 10.39	266.48 ± 6	8.84 ± 4.66	1.9 ± 0.1	318.33 ± 13	Square	612.67 ± 7.3	2.67
MN-D	300	390.96 ± 10.84	214.04 ± 17.2	19.11 ± 6.71	1.84 ± 0.2	179.88 ± 4.8	Square	405.99 ± 5.48	1.56
MN-E	170	182.15 ± 2.92	151.33 ± 11.6	5.43 ± 1.04	1.21 ± 0.1	891.4 ± 24	Square	716.83 ± 8.91	1.10
MN-F	100	262.83 ± 9.27	247.21 ± 6.7	9.09 ± 1.35	1.07 ± 0.1	493.13 ± 3.8	Square	731.24 ± 5.67	1.57
MN-G	260	241.49 ± 5.88	272 ± 7.7	8.65 ± 0.68	0.89 ± 0.1	504.26 ± 15.2	Square	762.18 ± 7.91	1.58
MN-H	145	188.66 ± 10.4	98.42 ± 4.7	18.16 ± 6.96	1.93 ± 0.2	575.08 ± 3.1	Triangular	563.22 ± 4.8, 326.53 ± 3.7	2.28
MN-I	315	207.76 ± 10.77	260.74 ± 9.8	69.38 ± 33.03	0.8 ± 0.1	168.01 ± 5.4	Square	452.11 ± 11.72	11.14
MN-J	225	223.4 ± 9.08	178.8 ± 10.4	24.92 ± 2.85	1.26 ± 0.1	461.28 ± 7.2	Square	442.77 ± 4.83	5.85

*Determined based on the distance between the base of needles

**Determined based on the distance of centre-to-centre between two adjacent needles in *x* (or *y*; result in bracket) directions

MNs are typically 100–1000 μm in height, with a base between 50 μm and 300 μm [45]. Although longer MN may counteract skin deformation to enable a reliable skin insertion and deeper drug delivery, shorter needle height can help to ensure that insertion remains largely painless. While a wider needle base increases mechanical strength and fracture force, it could also make the MNs more challenging to penetrate deeper.

Tip diameter is crucial for successful skin penetration. A majority of the MN patches have a tip diameter of < 25 μm (Table 3). Only MN-A and MN-I have larger tip diameters of 50.36 ± 6.36 μm and 69.38 ± 33.03 μm, respectively. MN array with tip diameter of 20–80 μm have been shown to be sufficiently sharp for effective skin penetration [44, 46]. Conversely, a reduced tip diameter has been reported to weaken the MN shaft, making the MN prone to fracture and thus impacting the skin insertion efficiency [47]. Overall, the MN patches are expected to be sufficiently sharp to penetrate the skin.

Most MN patches have an INS of ~ 320–686 μm with ~ 100–300 needles per patch. However, MN-E has the highest INS at 891.4 ± 24 μm, while MN-I exhibits the lowest (168.01 ± 5.4 μm), along with the highest needle count at 315 needles per patch. In contrast, MN-B has only 16 needles in its array and thus having among the highest INS (~ 700 μm). Most MN patches exhibited a pitch of ~ 406–763 μm, except MN-A and MN-B, whose pitch was 1301.98 ± 7.02 μm and 1335.9 ± 29.77 μm, respectively. This difference may be attributed to their fabrication methods, as discussed previously. Mechanically, skin behaves like a viscoelastic material and exhibits more elastic properties under light applied loads [48, 49]. Hence, the skin insertion of a MN array with high needle density and/or low INS/pitch is challenging and may require a higher force applied to achieve complete skin penetration [47, 50]. It is crucial to note that increasing INS can lead to an increased penetration efficiency [47, 50–52]. Hence, MNs with low needle counts and high INS should achieve a higher skin penetration efficiency even with a lower penetration force.

Apart from the dimensional measurements, various MN distribution patterns such as square, rectangular and triangular are crucial parameters because of their effects on buckling force, bending force and bending stress of MN patches [53]. Most MN patches featured a needle arrangement in a square pattern except for MN-H, which featured a triangular pattern. The preference for the square pattern may be due to its better skin piercing than other patterns [54].

### Mechanical properties of MN patches

An effective MN delivery system should exhibit adequate mechanical properties for reliable skin penetration and efficient drug delivery [55]. The mechanical strength of MN arrays is essential to prevent damage, including breaking and bending during usage or handling which would greatly limit their applications [23].

**Fig. 4:**
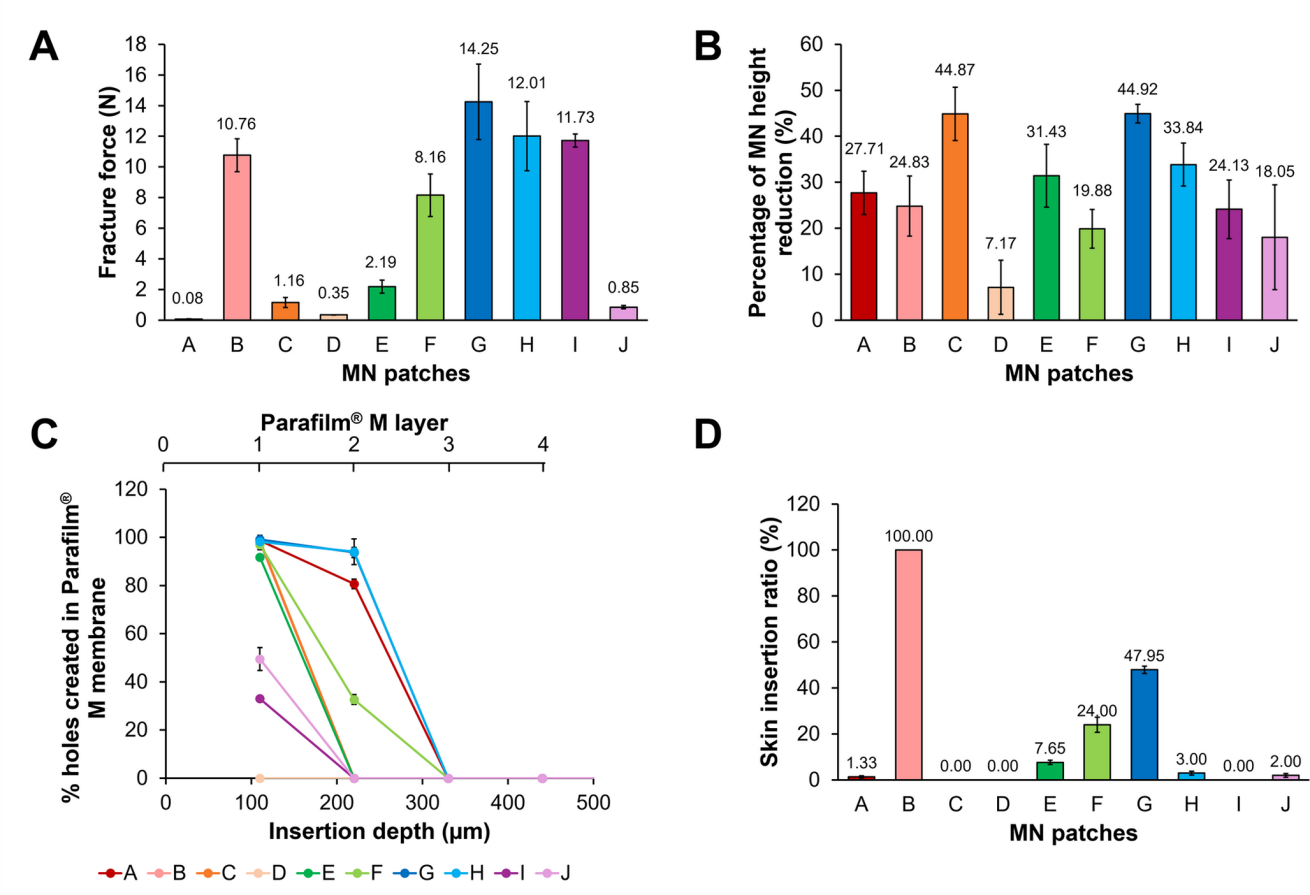
**(A)** Fracture force, **(B)** percentage of MN height reduction, **(C)** percentage of holes created in Parafilm^**®**^ M layers* and **(D)** porcine ear skin insertion ratio of MN patches (mean  ± SD, n = 3). *Profiles overlapping among MN-B, MN-C and MN-E as well as between MN-G and MN-H due to a similar penetration

Fig. 4 presents the mechanical evaluation of MN patches. MN-B, MN-G, MN-H and MN-I achieved among the highest fracture force of > 10 N in the axial compression test for whole MN arrays while the rest MNs showed a fracture force of < 3 N (Fig. 4A)

Upon observation of the MN after the axial compression test, some MNs appeared bent at the tip (MN-A, MN-B, MN-D, MN-E and MN-G) while other MNs (MN-C, MN-F, MN-H, MN-I and MN-J) collapsed (Figure S1 in Supplementary Material). The fracture force profiles (Figure S2 in Supplementary Material) showed a sudden drop in the force, with the highest peak force considered as the fracture force. It is noted that none of the MN patches has their baseplate broken. The different post-test appearance of needles may indicate the differences in their mechanical strength, probably due to the dimension and materials used. The collapsed MNs (MN-C, MN-F, MN-H, MN-I, and MN-J) had a narrower base width (< 265 μm) and were likely inherently soft due to the nature of their material. In contrast, the bent MNs appeared to have a wider and stronger base and shaft structure, contributing to their increased mechanical strength. About 70% of the MN patches underwent > 20% of MN height reduction (Fig. 4B) while MN-F and MN-J showed marginal MN height reduction at 19.89 ± 4.23% and 18.05 ± 11.41%, respectively. Only MN-D recorded a very low MN height reduction at 7.18 ± 5.9%. Peng, Vora, Domínguez-Robles, Naser, Li, Larrañeta and Donnelly [56] who fabricated poly(lactic-co-glycolic acid) MN patches with Plasdone^®^ K-90D (15%w/w) and glycerol (1.5%w/w) as base reported that MN patches that withstood 32 N force with a height reduction < 20% indicated non-fragile MN tips. Although this has been generally accepted to be a quick indicator for the needle strength in several reports [56–58], experimental variables such as the force applied and the test speed could affect the result differently.

It is important to highlight that the low MN height reduction observed in MN-D and the high fracture force recorded for MN-I could potentially be inaccurate. In the case of MN-D, the needles were found to rebound after compression upon observation during analysis which may be related to the elastic texture of the materials used. Additionally, as shown in Fig. 3, the needles for both MN types were not intact upon purchase, likely due to inadequate protection resulting from unsuitable packaging (Table 1). This may have led to the contribution of force from the MN baseplate, especially for MN-I.

No established standards currently exist for MN mechanical characterisation tests, largely due to the variations in experimental setups, equipment, MN materials and needle geometry [59, 60]. The choice of an appropriate test likely depends on the MN design, formulation and the scope of the investigation. Regardless of the test selected, the primary consideration remains achieving a reasonable safety factor or margin of safety to ensure successful skin penetration [23]. The safety factor is defined as the ratio of MN failure force to the skin insertion force [40, 61]. To minimise the risk of mechanical failure, MNs should achieve a safety factor ≥ 1 [23, 40]. Nonetheless, all MN patches reported a safety factor of < 1 with MN-G, MN-H and MN-I demonstrating the highest fracture force, corresponding to a safety factor of 0.71, 0.60 and 0.59, respectively with a force of 32 N applied.

Further scrutinising the data, we performed MRA to reveal the influence of MN dimensions on the mechanical parameters measured as shown in Table 4. The models agreed that tip diameter, aspect ratio and pitch have a prominent influence on both failure force and percentage of MN height reduction.

**Table 4:** MRA models predicting mechanical properties and insertion performance of MN patches (only significant values are shown)

MN related parameters	B, unstandardised coefficients			
	Failure force (*N*)	Percentage of MN height reduction (%)	Percentage of holes created in the first Parafilm^®^ M layer (%)	Percentage of holes created in the second Parafilm^®^ M layer (%)
Constant	20.853	-163.844	-224.246	
Height		0.008	-0.094	-0.558
Base width	-0.092			
Tip diameter	0.127	-1.201	-2.927	-2.855
INS	-0.038		-0.047	-0.287
Aspect ratio	-6.061	31.886	61.590	114.292
Pitch	0.064	0.171	0.374	0.547
Number of needles		0.159	0.101	0.397
Needle density		10.647	21.924	14.153
*R* ^*2*^	0.890	0.995	0.994	0.986
*p* value (ANOVA)	0.048	0.040	0.021	0.047

Specifically, increases in base width, INS and aspect ratio were associated with a reduction in failure force, whereas larger tip diameter and pitch contributed positively. Other variables did not significantly influence the failure force (*p* > 0.05). While, tip diameter is the only factor contributed negatively to the percentage of MN height reduction. Other parameters were consistently statistically insignificant, likely due to multicollinearity. Interestingly, aspect ratio and needle density (only for percentage of MN height reduction) appeared as the most crucial factor affecting both mechanical evaluations. This is in agreement with previous findings by Gittard, Chen, Xu, Ovsianikov, Chichkov, Monteiro-Riviere and Narayan [62] and Ando, Miyatsuji, Sakoda, Yamamoto, Miyazaki, Koide, Sato and Izutsu [63], showing an inverse relationship between mechanical strength and aspect ratio on 3D printed MNs and polyvinyl alcohol-based MNs, respectively.

### Insertion profiles of MN patches

Mechanical disruption of the SC is a fundamental function of MNs and, thus, puncture performance is also ranked as one of the most ‘critical’ and ‘prioritised’ quadrant in the CQA list for MN products aforementioned [27]. Together with physical stability, other physical attributes including the morphology, dimensions, arrangement of MN projections (such as height, shape, sharpness, and INS/pitch) and mechanical strength may also significantly influence the puncture performance [27]. Both in vitro and in vivo data are valuable for puncture performance.

The in vitro insertion performance of MN patches was first tested using Parafilm^®^ M as a validated skin simulant [23, 24]. Figure 4C shows that all MN patches, except MN-D successfully penetrated the first layer of the Parafilm^®^ M model. The failure of MN-D to penetrate the Parafilm^®^ M is not surprising due to the bent and deformed MNs as discussed previously. In the first layer, MN-A, MN-B, MN-C, MN-E, MN-F, MN-G and MN-H shared similar percentage of holes created (≥ 98%), while MN-I and MN-J recorded 33.02 ± 0% and 49.48 ± 4.77%, respectively. Besides, only 40% of the MN patches (MN-A, MN-F, MN-G and MN-H) penetrated the second layer (depth: ~ 220 μm). The penetration into the second layer was reduced with MN-H demonstrating the highest penetration of 94.03 ± 5.32% in the second layer. This is followed by MN-G, MN-A and MN-F, which achieved penetration of 93.72 ± 2.12%, 80.67 ± 2.01% and 32.67 ± 2.1%, respectively. However, none of the MN patches penetrated beyond the second layer. This is consistent with the measured MN heights as most MNs had a needle height < 300 μm. Despite having the tallest needles (504.98 ± 10.39 μm), MN-C only penetrated the first layer of the Parafilm^®^ M membrane. The poor in vitro insertion is likely due to its relatively weak needles with a fracture force of ~ 1 N. Individual in vitro insertion performance of MN patches can be found in Supplementary Material for a clearer presentation.

Theoretically, an increase in MN density may counteract the skin piercing ability of MN patches by reducing the pressure exerted at each MN tip, a phenomenon known as the ‘bed-of-nails’ effect [64]. The influence of needle density and the spacing between needles on the skin insertion ability has been widely investigated. Olatunji, Das, Garland, Belaid and Donnelly [50] reported a reduction in the insertion force from ~ 0.5 to ~ 0.1 N when increasing the INS from 30 to 600 µm using neonatal porcine skin ex vivo. However, the dependence of insertion force on INS diminished beyond 150 µm. Similarly, Kochhar, Quek, Soon, Choi, Zou and Kang [47] observed a ~ 20% increase in the percentage of needle penetration using both porcine ear skin and full thickness human cadaver skin ex vivo by doubling the MN pitch from 1200 to 2400 µm. A later computational study by Shu, Heimark, Bertollo, Tobin, O’Cearbhaill and Annaidh [52] also demonstrated that increasing INS provides more space for MNs to establish contact with skin surface, enhancing penetration efficiency.

In the present study, a moderate to strong correlation was observed between the space between needles and the percentage of holes created in the first layer of Parafilm^®^ M membrane (Pearson’s correlation; *R*^*2*^: 0.67 for pitch and *R*^*2*^: 0.65 for INS). Additionally, a moderate negative correlation was reported between needle density with the percentage of holes created in the first layer of Parafilm^®^ M membrane (Pearson’s correlation; *R*^*2*^: -0.52 for needle density). These findings align with previous reports indicating that the spacing between needles (INS or pitch) and/or needle density may affect MN penetration into the skin [47, 50, 64]. However, contradictory results have also been reported for MNs with INS of 250, 500 and 1000 μm [65], 30–600 μm [66] and MNs with 280–1314 needles/array [67]. Collectively, these reports suggested that other factors such as MN geometry, application force and use of applicator may influence MN puncture performance. The phenomenon is unsurprising, given the variability in the mechanical evaluation setups and the type of MNs studied.

In an attempt to further analyse the data for only the first two Parafilm^®^ M layers using MRA, the regression models (Table 4) agreed with the Pearson’s correlation results where pitch is one of the significant predictors. Together with aspect ratio, both two parameters which contributed significantly to the previous mechanical evaluations also appeared to substantially affect the in vitro insertion profiles. It is widely acknowledged that a longer needle with a wider gap (INS) is expected to penetrate deeper into the skin.

The in vitro skin insertion test provides a good indicator of the relative mechanical strength of MN patches. The skin insertion ratio ranges from ~ 1% to full insertion while 3 out of 10 MN patches (MN-C, MN-D and MN-I) exhibited no ex vivo insertion. Despite nearly full penetration of the first Parafilm^®^ M layer by 7 out of 10 MN patches, a complete in vitro porcine ear skin insertion was only demonstrated by MN-B and this was followed by MN-G (47.95 ± 1.58%), MN-F (24 ± 3.27%) and MN-E (7.65 ± 0.97%). Notably, MN-B can be said to fulfil the desired MN dimension and mechanical properties with the lowest needle density (0.2515 needles/patch area), the widest space between needles (INS: 685.6 ± 12.2 μm; pitch: 1335.9 ± 29.77 μm) and the smallest tip diameter (6.46 ± 2.31 μm). Previous studies have collectively reported that an increased spacing between needles (INS and pitch) can enhance penetration and reduce insertion force [47, 50, 52, 64]. In addition, a smaller tip diameter is particularly advantageous as this reduces the contact angle and insertion force required to penetrate the outer skin layer, minimising both pain and the risk of MN breakage [59]. Using logistic regression for the geometrical optimisation of MN eye patches, Lim, Tiew, Zhang, Ho, Kachouie and Kang [68] reported a substantial decrease in penetration efficiency from ~ 80% for MNs with a tip diameter of 50 and 100 μm to ~ 30% for those with a larger tip diameter (200 μm). Similarly, Römgens, Bader, Bouwstra, Baaijens and Oomens [69] observed that MNs with a tip diameter > 5 μm predominantly caused skin indentation, followed by a sudden insertion, while MNs with a tip diameter of 5 μm can be smoothly inserted into human skin ex vivo. A correlation analysis between fracture force and skin insertion revealed a strong positive relationship (Pearson’s correlation; *R²* = 0.64), suggesting that MNs with higher fracture forces are more likely to achieve higher skin insertion ratios. These findings supported the notion that the MN dimension is crucial in determining the mechanical properties and insertion performance of MNs.

**Fig. 5:**
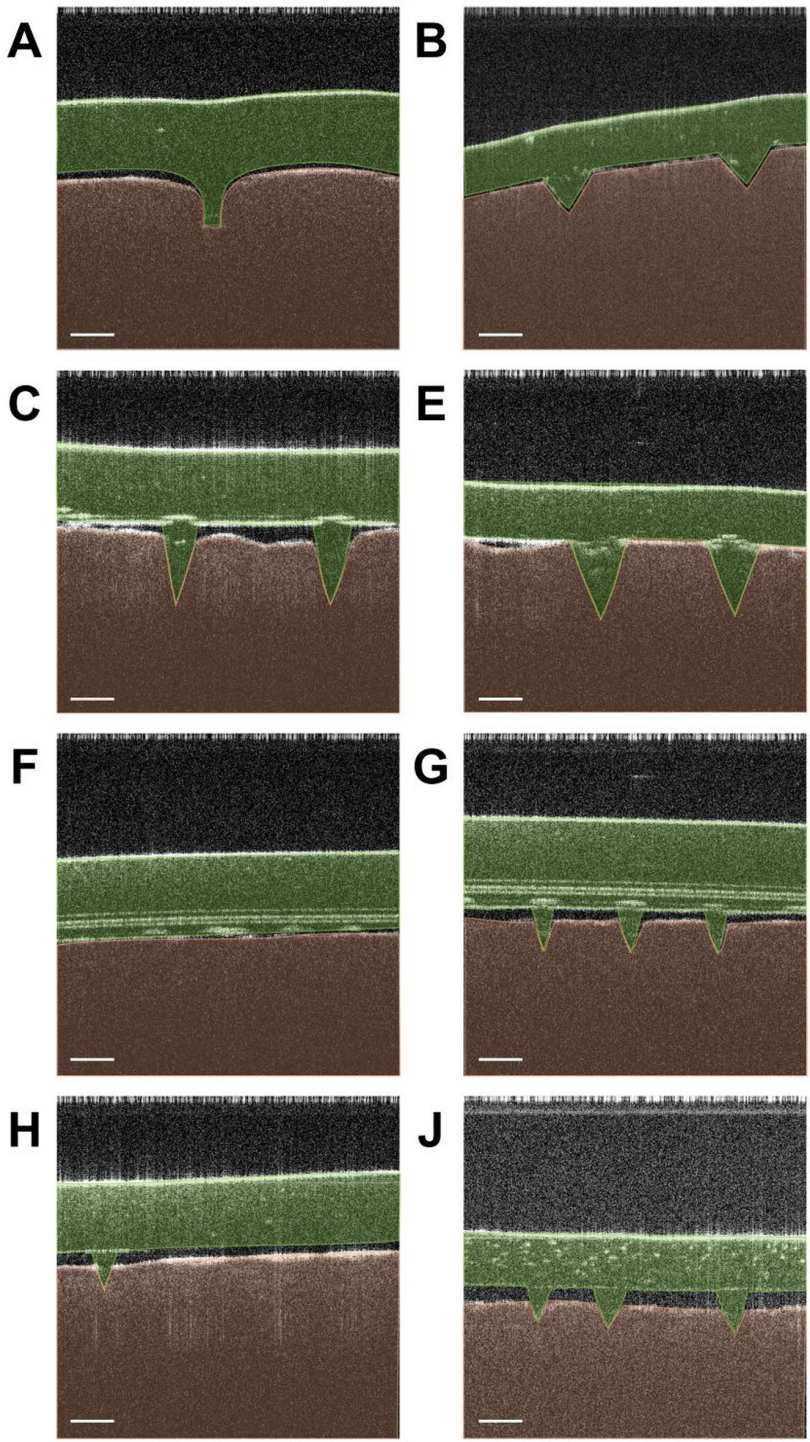
Representative OCT images of selected MN patches (alphabet represents MN code) following ex vivo porcine skin insertion (scale bar: 500 μm; orange colour: skin layer; green colour: inserted MN; original OCT images can be found in Figure S4 of Supplementary Material)

Further OCT analysis was used to visualise the MN skin insertion in real time. Figure 5 shows representative OCT images of MN patches following ex vivo porcine skin insertion. The original uncoloured OCT images were illustrated in Figure S4 (Supplementary Material). The images of MNs before and after OCT analysis can be found in Figure S5 (Supplementary Material). Most MNs were shown to pierce through the skin except MN-F while MN-D and MN-I were omitted from the test due to their poor Parafilm^®^ M and failed ex vivo porcine skin insertion. Nevertheless, MRA results were not significant to relate the MN dimensions on both ex vivo insertion results owing to the lack of data points for accurate predictions.

MN-B, MN-E and MN-G, which achieved among the highest Parafilm^®^ M insertion efficiencies (MN-B: ~ 98%; MN-E: ~ 92%; MN-G: ~ 99%) and skin insertion ratios (MN-B: 100%; MN-E: ~ 8%; MN-G: ~ 48%), demonstrated clear skin insertions with their MN structures embedded into the skin (Fig. 5B, E and G, respectively). In contrast, despite achieving nearly full insertion into the first layer of Parafilm^®^ M, MN-C failed to create any holes upon ex vivo skin insertion. However, it successfully penetrated the skin in the OCT analysis, alongside MN-A, MN-H and MN-J which exhibited a skin insertion ratio of ≤ 3%. The discrepancy between the ex vivo skin insertion test and OCT analysis observed for MN-C is likely due to the different sensitivities between the two techniques. The dye staining method relies on the formation of open and dye-permeable microchannels and skin tension changes during MN removal may cause biomechanical changes in the skin, resulting in a 0% insertion ratio. In contrast, OCT imaging can detect subtle structural changes beneath the skin surface in situ, capturing microchannels or skin deformation even when they are too small or transient to be stained. Thus, while dye staining did not indicate successful insertion, OCT analysis confirmed that MN-C was capable of penetrating the skin. This observation is consistent with previous reports highlighting that dye staining may underestimate microneedle insertion compared to more sensitive imaging techniques such as OCT [66, 70].

Surprisingly, MN-F showed no evidence of skin penetration under OCT analysis despite achieving Parafilm^®^ M insertion efficiency of ~ 97% (1st layer) and skin insertion ratio of ~ 24%. Given that OCT is a semi-quantitative imaging technique [71], the OCT test was repeated several times to confirm this result. There may be a possibility that the OCT failed to capture the tiny holes created in addition to the light attenuation due to a large gap between the patch and the skin. The failure of MN-F to breach the SC may also be attributed to its soft material nature, leading to the MN collapsing upon skin insertion, as observed in microscopic images after axial compression test (Figure S1 in Supplementary Material) and OCT analysis (Figure S5 in Supplementary Material).

Moreover, discrepancies between in vitro and ex vivo insertion behaviours may stem from the interplay between needle morphology and mechanical strength of the MN patches. The considerable variation in constituent materials and design of the MN patches may further contribute to these inconsistencies. While MNs create a nearly imperceptible micron-scale disruption of the outermost skin barrier, their functionality ultimately depends on their ability to effectively breach the skin, especially the SC. It is worth to mention that Parafilm^®^ M and the porcine skin have essentially different mechanical properties which also contributed to the different results here. Parafilm^®^ M has plastic behaviour which does not recover its shape when the force is removed and therefore any deformation made is permanent and cumulative. This suggested that Parafilm^®^ M can yield more easily under pressure. While, the skin is stronger and more elastic (elastic modules of Parafilm^®^ M: 170 MPa [72]; human skin: 5 kPa – 140 MPa [73]) which may deflect the energy and recover after the force is removed after patch insertion.

To date, there are no standard guidelines from regulatory authorities or international pharmacopoeias for MN characterisation and device production [74], despite efforts by the Regulatory Working Group which has identified a list of potential CQAs [27]. The variability observed in in vitro and ex vivo puncture performance tests highlights the need for multiple complementary testing methods. This supports the idea of using different puncture performance tests to ensure reliable MN skin insertion where in vitro testing may primarily serve preclinical development, while ex vivo and in vivo tests are necessary to establish in vitro-in vivo correlation (IVIVC) and shall be replaced solely by the in vitro tests [27]. Therefore, establishing a discrete and quantitative measure of puncture efficiency should be prioritised to ensure standardised assessment of MN performance [27].

## Conclusion

The functionality of MN products relies heavily on the capability of the MN in breaching the SC barrier and creating microchannels. This study presents a comprehensive evaluation of the integrity, mechanical and skin penetration performance of commercial MN patches, offering critical insights into the MN product quality. While most MN patches achieved nearly full penetration in the in vitro Parafilm^®^ M model, their ex vivo skin insertion performance was inconsistent, particularly in the OCT analysis. This variability is influenced not only by material properties and the skin models but also by correlations observed between MN dimensions, mechanical properties and in vitro/ex vivo insertion profiles. These findings suggested that successful skin penetration requires an optimal balance of mechanical strength and needle morphology to maintain MN structural integrity. At the same time, this raises an alarm for MN manufacturers to prioritise the product quality and assurance throughout the product development process.

When assessing the dimensions of MN patches, we found that their integrity is significantly influenced by the container closure/packaging. Proper packaging plays a crucial role in maintaining the structural stability of MN patches, ensuring they remain intact and effective throughout storage, transport and handling. Given the delicate nature of MNs, exposure to environmental factors such as humidity, temperature fluctuations, and mechanical stress can compromise their mechanical strength, leading to deformation or breakage. Packaging serves as the first line of defence against these risks by providing a controlled environment that preserves the physical and functional properties of MN patches. A well-designed container, especially with blister pack as primary packaging, not only protects the MNs from external pressures but also prevents potential contamination, ensuring the safety and efficacy of the MN products. This is particularly critical for maintaining the puncture efficiency of MN products, as any compromise in their integrity can reduce their ability to effectively penetrate the skin.

We believe that MN products represent an important ‘innovation’ that warrants greater attention, particularly regarding the preservation of MN integrity. It is crucial to recognise that regulatory frameworks, both national and global, should be harmonised to ensure consistent product quality for end users. We have highlighted two key aspects previously – the puncture performance of MNs and the packaging, both of which critically influence the overall effectiveness of MN products. These factors must be strongly prioritised during product development as without effective skin penetration, MN products risk being dismissed as a passing trend (‘fad’) as demonstrated in this study.

The important findings from current cosmetic MN products provide valuable insights, particularly for pharmaceutical MN products, which require stricter quality assurance before being released to the market. Unlike cosmetic MN patches, pharmaceutical MNs often carry active ingredients that must be delivered precisely and consistently. Any compromise in packaging can result in variations in drug delivery performance, potentially affecting therapeutic outcomes.

## Electronic supplementary material

Below is the link to the electronic supplementary material.

Supplementary Material 1

## Data Availability

The authors confirm that the data supporting the findings of this study are available within the article and its supplementary materials.
