# Supplementary material for: Assessing the integrity and mechanical properties of commercial microneedles: innovation or fad?
Source: Drug Deliv Transl Res. 2025 May 31;15(11):3986–4003. doi: 10.1007/s13346-025-01888-8 (PMC12507953; doi:10.1007/s13346-025-01888-8)
Supplement: Supplementary file 1 — Supplementary Material 1 [file 13346_2025_1888_MOESM1_ESM.docx]

**Supplementary Material**

**Assessing the integrity and mechanical properties of commercial microneedles: Innovation or fad?**

Jing Yi Lee^1^, Shi Hui Dong^1^, Keng Wooi Ng^2, 3^, Choon Fu Goh^1^*

*^1^*Discipline of Pharmaceutical Technology, School of Pharmaceutical Sciences, Universiti Sains Malaysia, 11800 Minden, Penang, Malaysia.

^2^School of Pharmacy, Faculty of Medical Sciences, Newcastle University, Newcastle upon Tyne, NE1 7RU, UK

^3^Translational and Clinical Research Institute, Faculty of Medical Sciences, Newcastle University, Newcastle upon Tyne, NE1 7RU, UK

* Correspondence: Choon Fu Goh, Discipline of Pharmaceutical Technology, School of Pharmaceutical Sciences, Universiti Sains Malaysia, 11800 Minden, Penang, Malaysia. Email: choonfugoh@usm.my


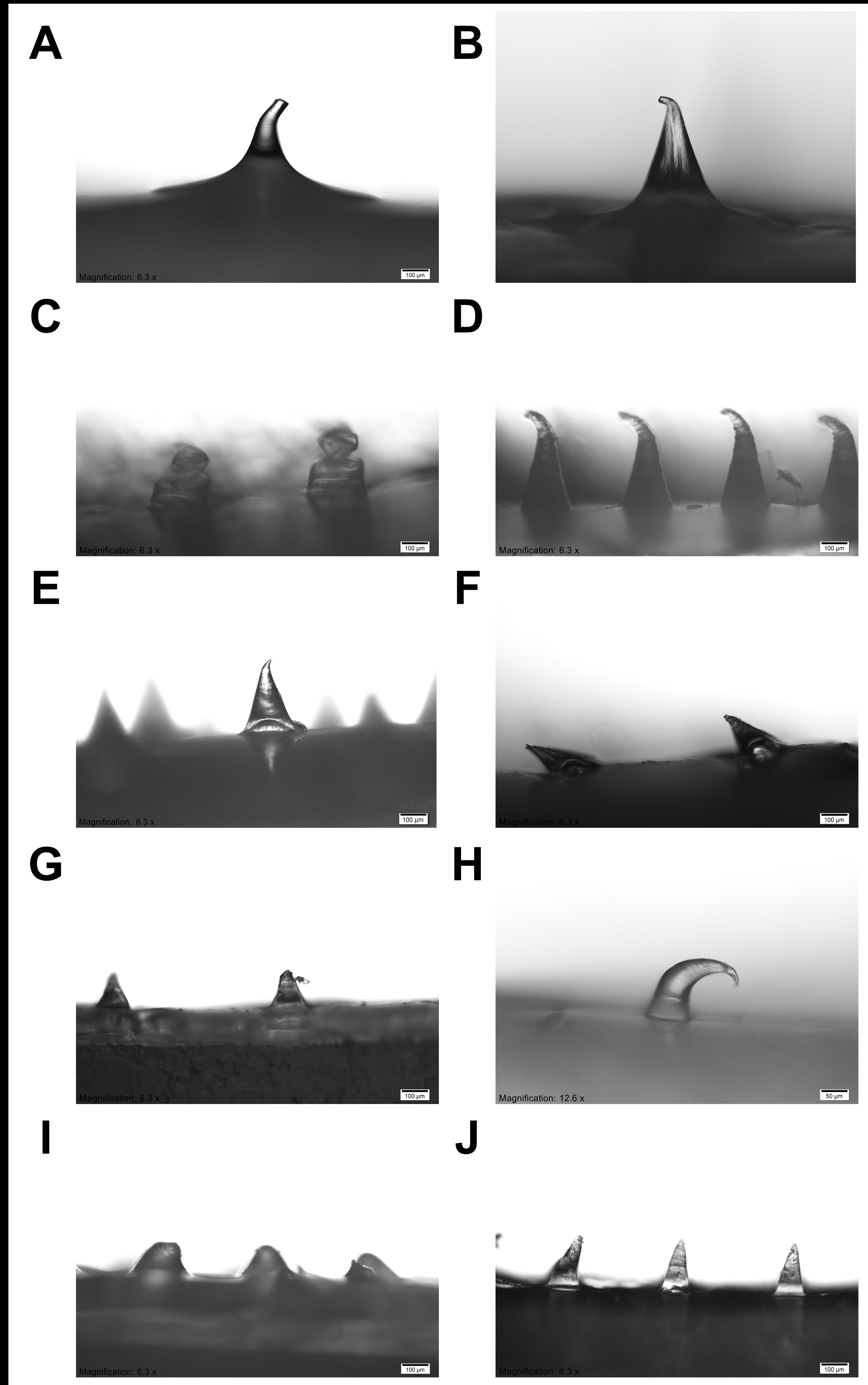


Figure S1 Microscopic images of MN patches (MN-A – MN-J) after axial compression test at a magnification of 6.3× (scale bar: 100 µm)


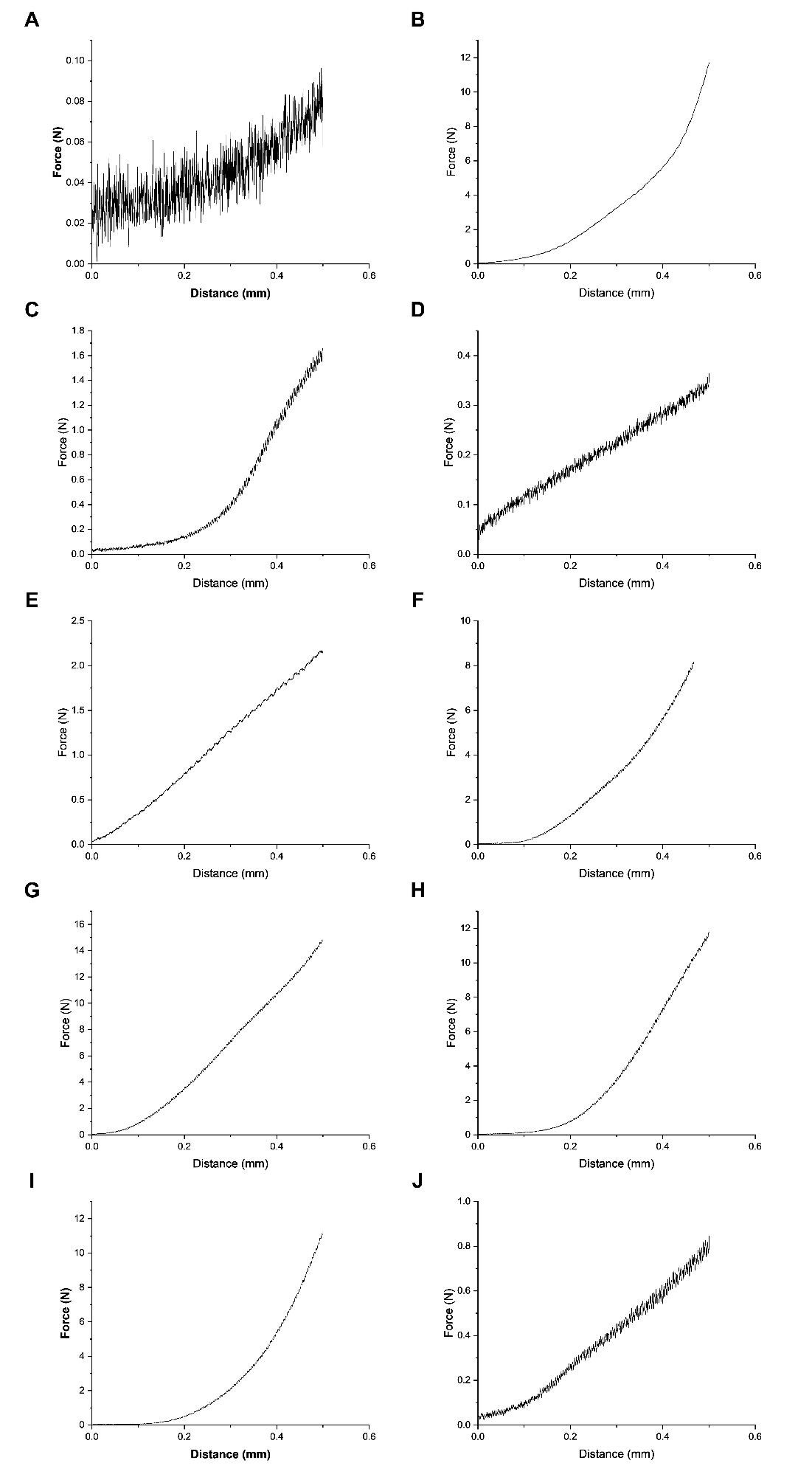


Figure S2 Force–displacement curves of MN patches after axial compression test


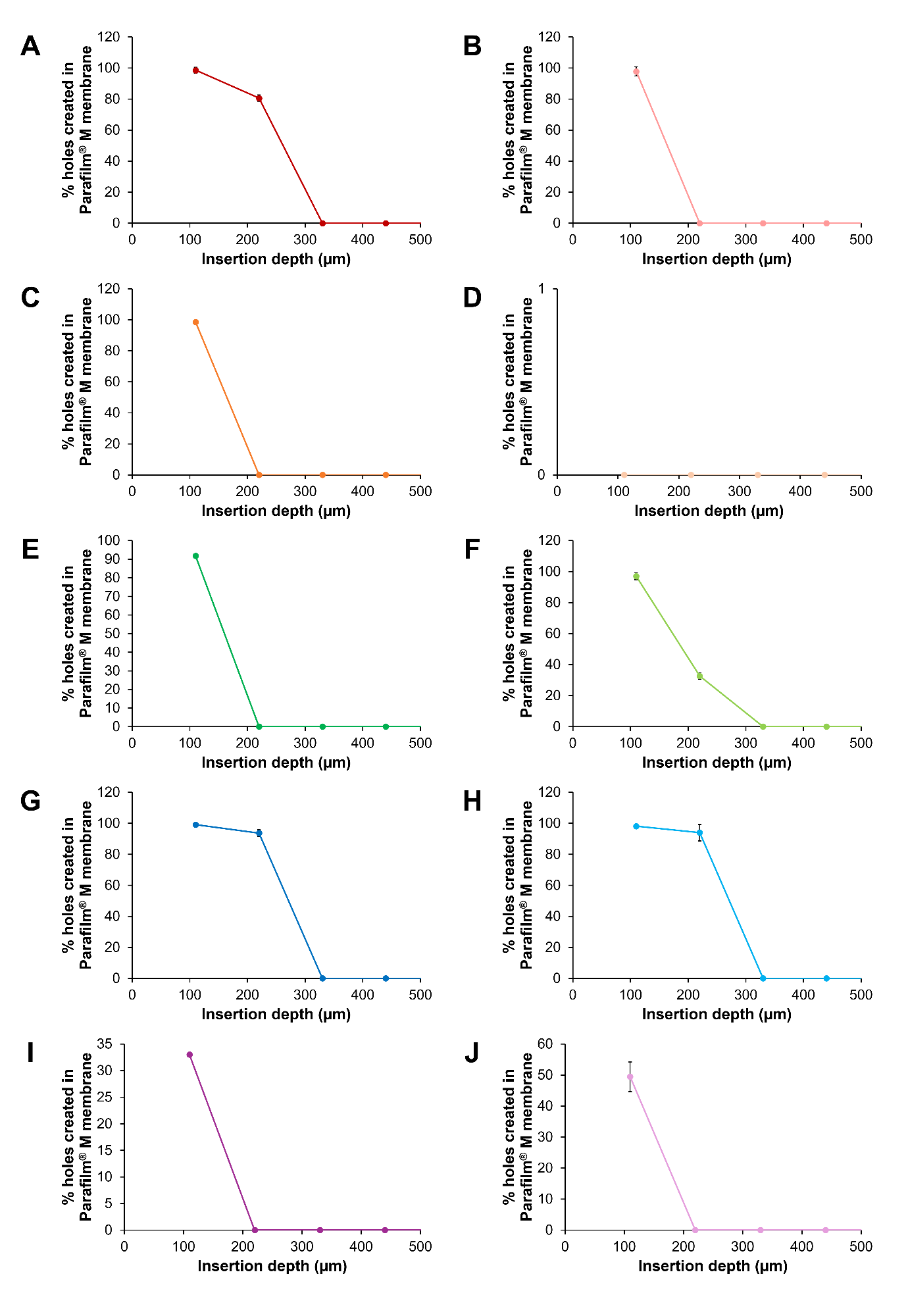


Figure S3 Percentage of holes created in Parafilm^®^ M layers of each MN patches (mean ± SD, n = 3).


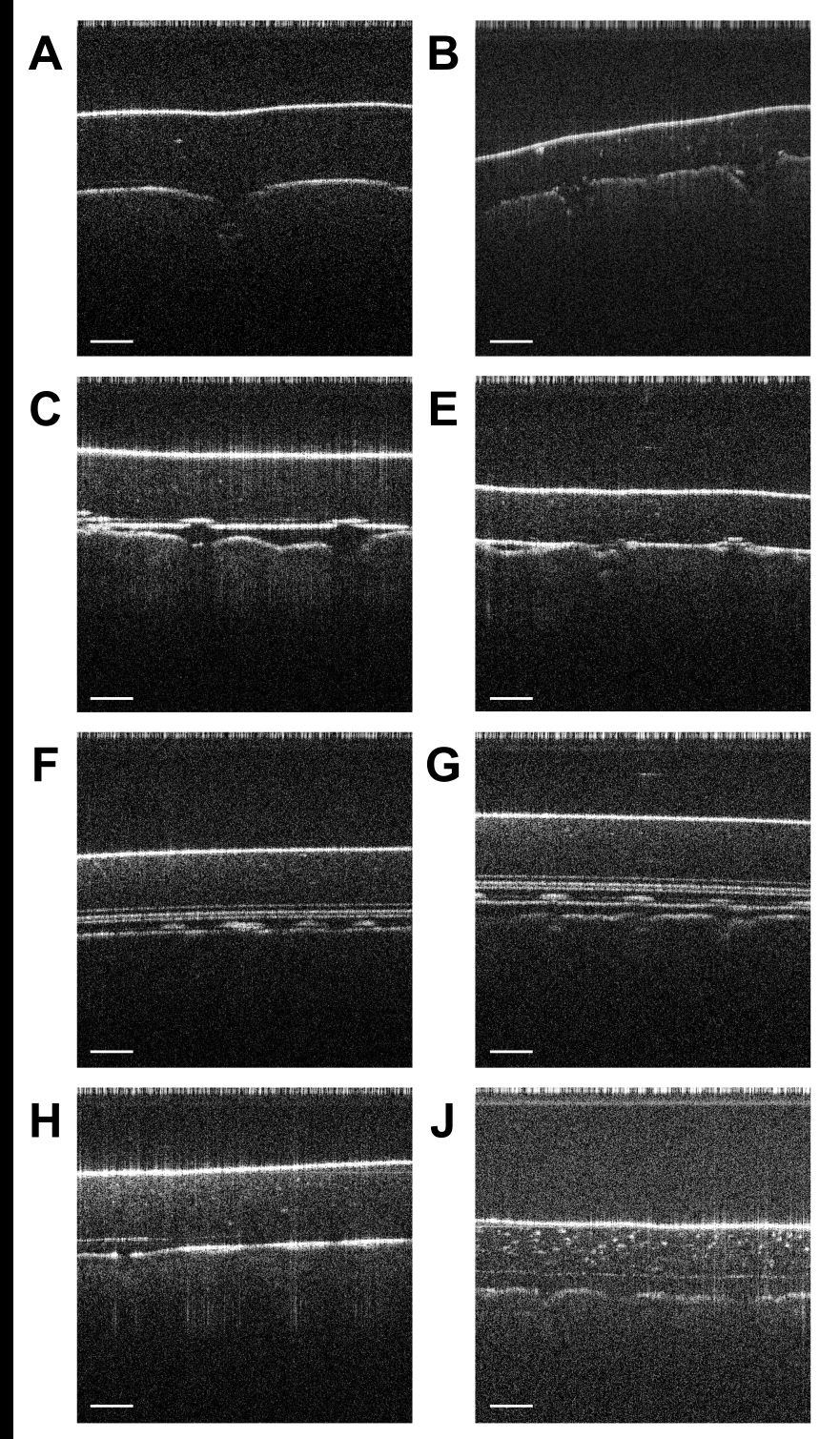


Figure S4 Representative OCT images of selected MN patches following their application into porcine skin *ex vivo* (scale bar: 500 µm)


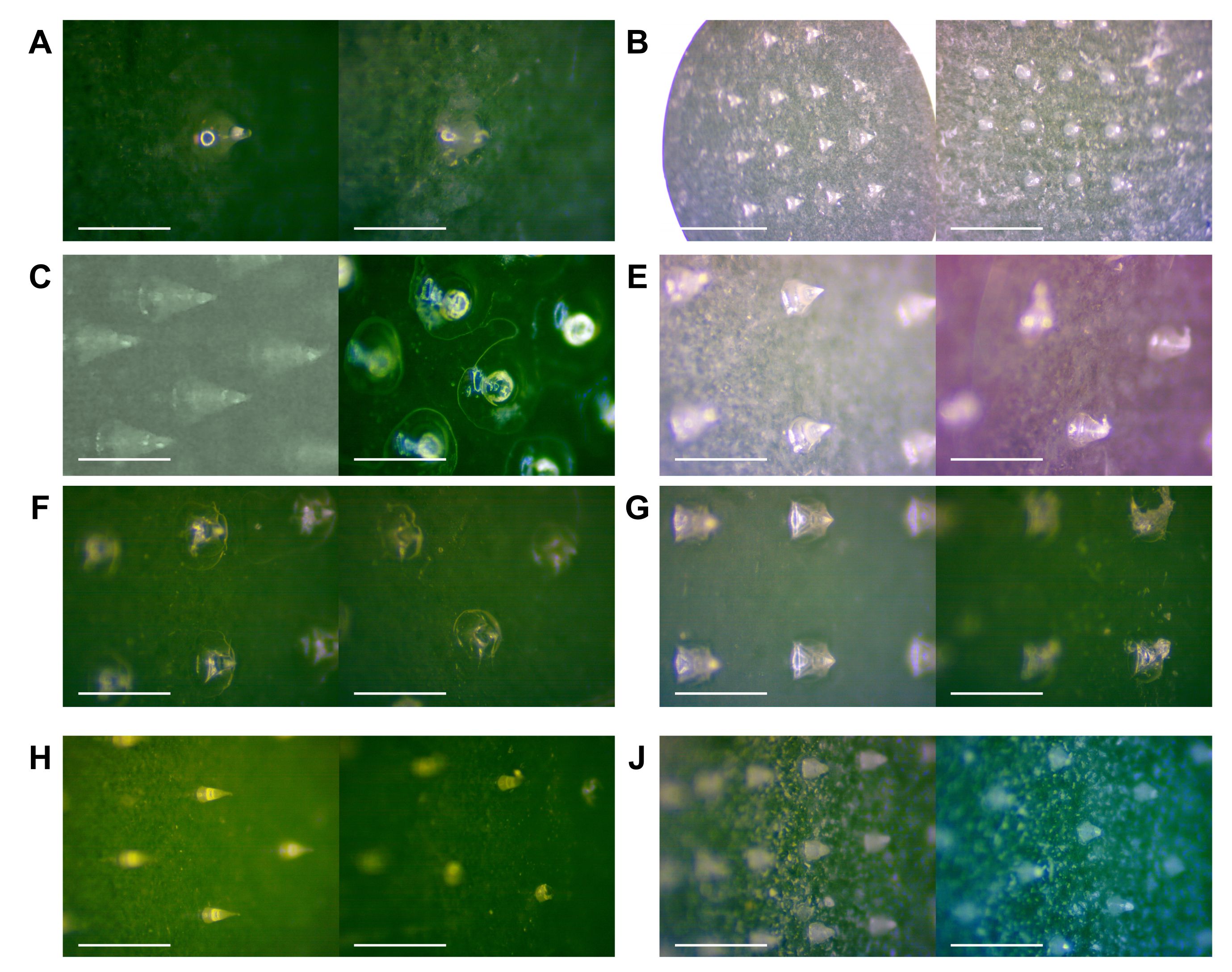


Figure S5 Microscopic images of selected MN patches before (left panel) and after (right panel) *ex vivo* skin insertion during OCT analysis (magnification: 3×; scale bar: 500 µm)
